# Challenges in Device Closure of Secundum Atrial Septal Defect in Older Patients in Their Fifth Decade and Beyond

**DOI:** 10.7759/cureus.22480

**Published:** 2022-02-22

**Authors:** Anil K Singhi, Soumya K Mahapatra, Dilip Kumar, Somnath Dey, Amiya Mishra, Arnab De

**Affiliations:** 1 Pediatric Cardiology, Medica Super Specialty Hospital, Kolkata, IND; 2 Cardiology, Medica Super Specialty Hospital, Kolkata, IND; 3 Cardiac Anesthesia and Critical Care, Medica Super Specialty Hospital, Kolkata, IND; 4 Anesthesia, Medica Super Specialty Hospital, Kolkata, IND

**Keywords:** stroke, bleeding, diastolic dysfunction, arrhythmia, pulmonary hypertension, challenge, device closure, older patients, atrial septal defect

## Abstract

Objective

Transcatheter atrial septal defect (ASD) device closure in the older population presents a greater challenge due to the long-standing effect of atrial left-to-right shunt. This study analyzes the challenges encountered in transcatheter ASD device closure in older patients in their fifth decade and beyond.

Methods

Adults aged 40 years and above with significant secundum ASD who underwent transcatheter ASD device closure between June 2015 and April 2021 were analyzed. Challenges were classified as major and minor challenges based on their impact on the alteration of the treatment protocol. Patients were categorized into three subgroups according to age. Group 1 consisted of patients aged 40-49 years (n = 13), Group 2 consisted of patients aged 50-59 years (n = 16), and Group 3 consisted of patients aged 60 years and above (n = 8).

Results

A total of 37 patients were analyzed. The challenges encountered were arrhythmia, pulmonary hypertension, left ventricular diastolic dysfunction, bleeding, stroke, coronary artery disease (CAD), hypertension, and airway disease. Thirteen percent of challenges were seen in pre-procedure time, whereas 79% of challenges during the procedure and 8% of challenges during post-procedure were seen. Thirty-five patients (94.6%) underwent transcatheter ASD device closure. Two patients (5.4%) did not undergo transcatheter ASD device closure due to severe diastolic dysfunction and associated CAD, respectively. Eleven major challenges were encountered in 10 patients in which one patient had a dual challenge of bleeding and arrhythmia. Thirteen patients (35.1%) had smooth procedures without any challenges encountered. Twenty-seven minor challenges were encountered in 20 patients with some patients having an overlap of multiple major and minor challenges. The patients were doing well at the mean follow-up of 28 months.

Conclusions

Transcatheter ASD device closure in older patients who are 40 years and above is safe and effective. Such high-risk patients are prone to various challenges that can be effectively managed if optimally monitored on the basis of a proper understanding of the altered physiology and anticipation of the deviated course at various stages of the procedure.

## Introduction

Atrial septal defect (ASD) is one of the most common congenital heart diseases in the adult population. The estimated prevalence of this defect in adults is 0.88 per 1000 patients [1]. The natural history of an unoperated ASD is the onset of right heart dysfunction, pulmonary hypertension, and atrial arrhythmia in the fourth decade of life [2].

Transcatheter closure has become the preferred strategy for the treatment of secundum ASD due to its relatively high efficacy, lower morbidity, and low complication rate compared to surgery [3]. The success of this minimally invasive procedure has extended the feasibility of this procedure to high-risk older patients often accompanied by comorbidities. However, complications in this procedure are bound to occur in view of the effects of long-standing atrial level shunt and age-related cardiovascular and respiratory risk factors. Indeed, earlier studies have described the outcomes of transcatheter ASD closure patients in older patients. However, these reports included mainly middle-aged patients rather than a cohort of patients from fifth to seventh decades and beyond [4].

Some studies have drawn attention to the success of this procedure in the high-risk elderly patient subgroup [5,6], whereas other studies have outlined only peri-procedural and post-procedural complications rather than drawing focus toward pre-procedural risk factors influencing the procedural success and the outcome [3]. Pre-procedural holistic assessment of the risk factors that could pose challenges in the procedure determines and influences the choice of intervention as well as the course and outcomes of the intervention. Failure to acknowledge these factors can result in dire prognostic implications. Literature detailing pre-, peri-, and post-processional challenges of transcatheter ASD device closure in the older population is limited. Against this background, this study analyzed the challenges encountered in the transcatheter closure of secundum ASD in older adult patients aged 40 years and above.

## Materials and methods

Study design and patient population

A retrospective, observational, investigator-initiated study was conducted at a tertiary-care center in India from June 2015 and April 2021. All patients aged 40 years and above with a secundum ASD with significant left-to-right shunt undergoing device closure of the secundum ASD were studied. Patients with primum ASD, sinus venosus ASD, significant additional congenital heart defects, and associated myocardial disease were excluded from the study. The term “older” patients in the index study meant patients aged 40 years and above. Patients were categorized into three subgroups according to age. Group I consisted of patients in their fifth decade of life (n = 13). Group II consisted of patients in their sixth decade of life (n = 16). Group III consisted of patients in their seventh decade of life and beyond (n = 8). All patients provided informed consent for the procedure and subsequent data collection and analysis for the research purposes as general informed consent protocol of the hospital. The study protocol was approved by the Institutional Ethics Committee of Medica Super Specialty Hospital (Letter number: CERC/2021/jun/iv). The ethics committee waived off the requirement for patient consent for the retrospective record-based study. All patient information was anonymized to hide the identity of the subject.

ASD device closure

All patients underwent detailed clinical evaluation, oxygen saturation checking, chest x-ray, and echocardiogram. Detailed transthoracic echocardiography (TTE) and transesophageal echocardiography (TEE) were performed to delineate the anatomy of the defect and assess the suitability of transcatheter device closure of the defect. Operability was assessed clinically by the presence of cardiomegaly, flow murmur, tricuspid mid-diastolic murmur supported by chest x-ray, electrocardiography, and echocardiography. Cardiomegaly as evidenced on chest x-ray and increased pulmonary blood flow were considered as radiological markers for the significant left-to-right shunts. On echocardiography, significant left-to-right shunt across the ASD was confirmed by dilation of the right heart chambers along with significant diastolic flow across the tricuspid valve and brisk flow through the pulmonary veins.

Standard protocol for all ASD device closure procedures was patient admission one day prior to the intervention, intervention on the second day, and discharge on the third day. All patients underwent cardiac catheterization and coronary angiography (CAG) during the procedure. Pulmonary artery and left ventricular end-diastolic pressures (LVEDP) were measured in all patients in the cardiac catheterization. Peak pulmonary pressure ranging 35-50 mmHg was defined as mild pulmonary hypertension, 50-70 mmHg was defined as moderate pulmonary hypertension, and >70 mmHg was defined as severe pulmonary hypertension [7].

Systolic pulmonary artery pressure was used in the classification of different grades of pulmonary hypertension as the cohort had increased pulmonary blood flow-related elevation of pulmonary artery pressure. Pulmonary blood flow and pulmonary vascular resistance were assessed in patients who had moderate to severe pulmonary hypertension on direct pressure estimation. Pulmonary blood flow and resistance were not measured in patients with normal and mildly elevated pulmonary artery pressure with evidence of significant left-to-right shunt. LVEDP of 15 mmHg and above was considered as elevated levels [8]. Left ventricular end-diastolic pressure was assessed in all patients at baseline, and a value ≥ 15 mmHg was considered as elevated. Patients who had concerns of left ventricular diastolic dysfunction were scheduled for ASD occlusion with a balloon or device. LVEDP was reassessed 15 minutes after occlusion of the ASD. Intravenous furosemide was administered to patients with elevated LVEDP. The pre-procedure optimization of blood pressure was done where applicable. Amplatzer™ (Abbott, Plymouth, Minnesota) and Lifetech (LifeTech Scientific, Shenzhen, China) devices were deployed in patients requiring devices up to 40 mm. Patients requiring devices beyond 40 mm size had Lifetech devices implanted in view of size restriction in Amplatzer devices.

Challenges

Challenges were issues related to long-standing atrial shunts, procedures, or issues linked to advanced age. These factors directly or indirectly influence the procedure and require additional therapeutic intervention. The challenges were present before the intervention at the planning stage or during or after the procedure. These were classified as major and minor. Major challenges altered the routine treatment protocol, incurred extended stay and intervention, or necessitated readmission. Minor challenges were issues that had not required major alteration in the standard treatment protocol. Administration of adenosine or beta-blockers during the intervention to control transient arrhythmia or antihypertensive medication was considered as a minor intervention, which had not altered the standard protocol of care. The anatomical challenges in ASD closure such as deficient or floppy rims or multiple defects were independent of age. Hence, these factors were observed in the study but not analyzed as age-related challenges.

Statistical analysis

Continuous variables are expressed as mean ± standard deviation and categorical variables as frequency and percentage. The distribution of continuous variables within age groups was assessed by the Shapiro-Wilk tests for normal distribution. Continuous variables were compared between three age groups by one-way analysis of variance (ANOVA) or Kruskal-Wallis test as applicable. Due to the small number of multiple comparisons, the Bonferroni test was performed to determine the significance of pairwise differences in groups. A p-value < 0.05 was considered statistically significant. All data were analyzed with the SPSS Statistics (Statistical Package for the Social Sciences) for Windows, version 20.0 (IBM Corp., Armonk, NY). A chi-square test was performed to assess the association between gender and age groups, and a p-value of 0.014 was considered statistically significant. Weight was compared using one-way ANOVA, and a p-value of 0.775 was considered statistically insignificant. Continuous and categorical variables were interpreted in a similar manner.

## Results

**Figure 1:**
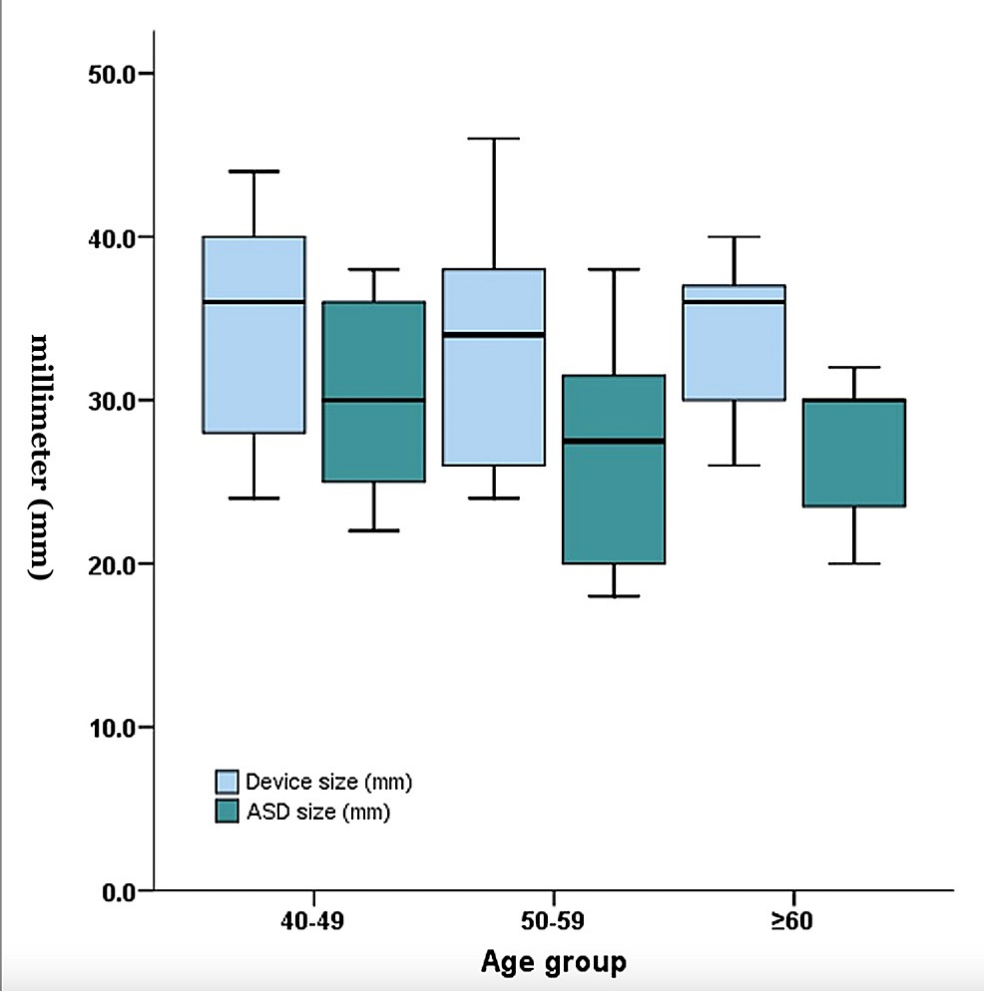
Box and whisker chart showing atrial septal defect size (green box) and defect device size (blue box) plotted in the y-axis for different age groups shown in the x-axis

**Figure 2:**
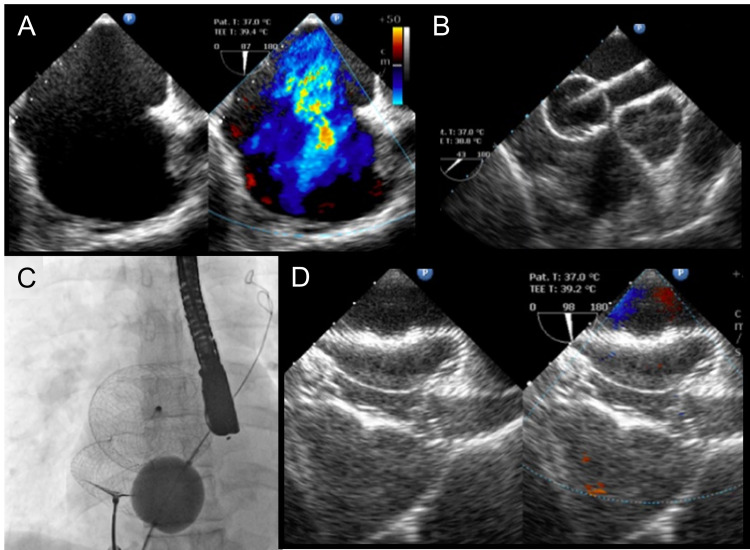
(a) Transesophageal echocardiography (TEE) with color Doppler showing a very large atrial septal defect with left-to-right shunt and thin and floppy posterior inferior margin. (b) TEE showing Equalizer balloon across the defect. (c) Fluoroscopic image showing balloon-assisted device closure of the defect. (d) TEE with color Doppler showing good device occlusion.

A total of 37 patients were analyzed (Table 1). Of the 37 patients, 35 (94.6%) underwent successful ASD closure. Two patients (5.4%) did not undergo device closure. One patient had significant double-vessel coronary artery disease (CAD) and underwent surgery as per the patient’s preference. The second patient had very significant ventricular diastolic dysfunction with intolerance to the balloon occlusion test. The mean ASD size was 28 mm, and the mean device size was 34 mm; 12 patients had device size ≥ 38 mm (Figure 1). Fifteen patients had a large ASD with floppy margins and aneurysmal interatrial septum. These patients required balloon-assisted ASD closure with a 33-mm Equalizer balloon (Figure 2). All patients except two patients with multiple defects had no residual flow after ASD device closure. Two patients with multiple ASD had placement of a large device in the bigger ASD. There was only mild residual flow through the small hole.

**Table 1:** Demographic and clinical characteristics of the patients All variables are expressed as n (%) or mean ± SD. *p-value is statistically significant. #p-value from one-way analysis of variance (ANOVA). PASP, Pulmonary artery systolic pressure; PADP; pulmonary artery diastolic pressure; PAP, pulmonary artery pressure; PAH, pulmonary hypertension; PVRI, pulmonary vascular resistance index; CAG, coronary angiography; CAD, coronary artery disease; ASD, atrial septal defect; LVEDP, left ventricular end-diastolic pressures; Qp:Qs, the ratio of pulmonary and systemic blood flow.

Variables	Patients (n = 37)	Group I (40–49) (n = 13)	Group I (50–59) (n = 16)	Group III (>60) (n = 13)	p-value
Age, years	52.1 ± 8.3	43.2 ± 3.1	53.5 ± 2.7	64.0 ± 3.1	<0.001*
Female	28 (75.7%)	13 (100%)	11 (68.7%)	4 (50.0%)	0.014*
Male	9 (24.3%)	0 (0.0%)	5 (31.3%)	4 (50.0%)	
Weight, kg	58.8 ± 13.6	59.0 ± 14.0	59.9 ± 10.9	55.7 ± 18.7	0.775^#^
Fluoroscopy time, mins	10.6 ± 7.7	13.2 ± 11.8	9.1 ± 3.86	9.5 ± 3.1	0.824
Pulmonary pressure and hemodynamic variables					
Pre-PASP, mmHg	37.1 ± 12.4	37.7 ± 13.8	35.2 ± 8.6	39.8 ± 17.0	0.985
Pre-PADP, mmHg	15.08 ± 4.6	16.9 ± 5.3	14.2 ± 3.7	13.9 ± 4.8	0.387
Pre-mean PAP, mmHg	23.1 ± 7.0	24.5 ± 8.4	21.8 ± 5.3	23.6 ± 8.2	0.813
Basal normal PAP (PASP < 35 mmHg)	21 (56.8%)	7 (53.8%)	9 (56.2%)	5 (62.5%)	0.416
Basal mild PAH (35–50 mmHg)	8 (21.6%)	3 (23.1%)	5 (31.2%)	0 (0.0%)	
Basal moderate PAH (50–70 mmHg)	7 (18.9%)	3 (23.1%)	2 (12.5%)	2 (25.0%)	
Basal severe PAH (>70 mmHg)	1 (2.7%)	0 (0.0%)	0 (0.0%)	1 (12.5%)	
Qp:Qs in moderate-severe group	2.6 ± 0.6	2.7 ± 0.3	2.0 ± 0.73	2.8 ± 0.7	0.405
PVRI in moderate-severe group, Wu.m^2^	2.5 ± 1.3	3.1 ± 0.8	1.3 ± 0.3	2.7 ± 1.7	0.119
Pre-LVEDP	13.0 ± 3.1	14.0 ± 3.7	12.3 ± 2.6	12.6 ± 2.9	0.223
Basal LVEDP (<15 mmHg)	29 (78.4%)	9 (69.2%)	14 (87.5%)	6 (75.0%)	0.456
Basal LVEDP (>15 mmHg)	8 (21.6%)	4 (30.8%)	2 (12.5%)	2 (25.0%)	
Post-LVEDP	14.07 ± 3.9	14.8 ± 1.7	14.4 ± 6.0	12.9 ± 2.4	0.154
Post-LVEDP (<15 mmHg)	18/27 (66.7%)	5/9 (55.6%)	6/10 (60.0%)	7/8 (87.5%)	0.351
Post-LVEDP (>15 mmHg)	9/27 (33.3%)	4/9 (44.4%)	4/10 (40.0%)	1/8 (12.5%)	
Coronary angiography					
Normal CAG	26 (70.3%)	11 (84.6%)	11 (68.7%)	4 (50.0%)	0.311
Minor CAD	10 (27.0%)	2 (15.4%)	4 (25.0%)	4 (50.0%)	
Major CAD	1 (2.7%)	0 (0.0%)	1 (6.2%)	0 (0.0%)	
ASD analysis					
Number of patients device closure done	35 (94.6%)	13 (100%)	14 (87.5%)	8 (100%)	0.495
ASD size, mm	28 ± 6.1	30 ± 6.0	26 ± 6.8	27 ± 4.4	0.279
Device size, mm	34 ± 6.6	35 ± 7.0	32 ± 7.2	34 ± 5.2	0.658
ASD device size < 38 mm	23/35 (65.7%)	7/13 (53.8%)	10/14 (71.4%)	6/8 (75.0%)	0.599
ASD device size > 38 mm	12/35 (34.3%)	6/13 (46.2%)	4/14 (28.6%)	2/8 (25.0%)	
Multiple defects	2/35 (5.7%)	2/13 (15.4%)	0 (0.0%)	0 (0.0%)	0.159
Assisted ASD closure	15 (40.5%)	7 (53.8%)	5 (31.3%)	3 (37.5%)	0.472
Device					
Amplatzer	11/35 (31.4%)	2/13 (15.4%)	7/14 (50.0%)	2/8 (25.0%)	0.150
Lifetech	24/35 (68.6%)	11/13 (84.6%)	7/14 (50.0%)	6/8 (75%)	
Anticoagulation/antiplatelet (post device)	35 (94.6%)	13 (100%)	14 (87.5%)	8 (100%)	0.495
Aspirin only	7 (0.2%)	2 (15.4%)	4 (25.0%)	1 (12.5%)	0.757
Dual platelet therapy	14 (0.4%)	4 (30.8%)	6 (37.5%)	4 (50.0%)	0.694
Antiplatelet with warfarin	14 (0.4%)	7 (53.8%)	4 (25.0%)	3 (37.5%)	0.318
Follow-up in months	28.0 ± 23.0	16.6 ± 20.0	38.9 ± 23.8	24.8 ± 17.4	0.028*

**Figure 3:**
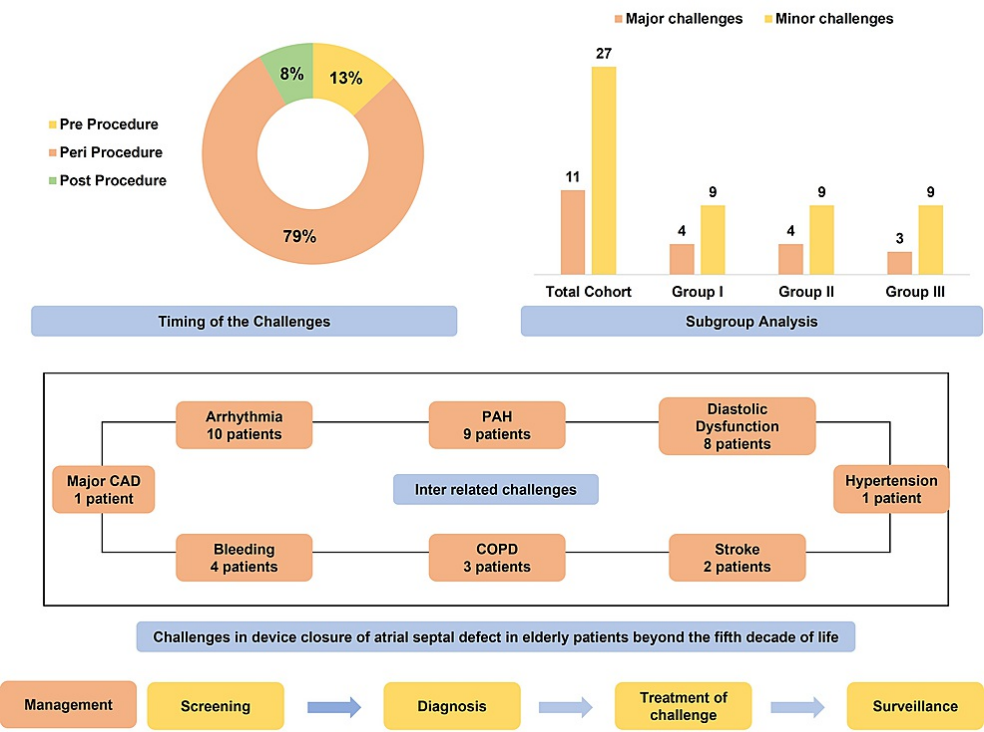
Major and minor challenges occurring in transcatheter atrial septal defect closure in elderly patients belonging to different age groups and management of the challenges

Various challenges were encountered during different stages of intervention. Thirteen percent of challenges were seen in pre-procedure time, whereas 79% of challenges during the procedure and 8% of challenges during post-procedure were seen (Table 2, Figure 3). Eleven major challenges were encountered in 10 patients. Two major challenges were encountered in one patient in the form of arrhythmia and bleeding. Challenges were equally distributed in all subgroups. Thirteen patients had smooth procedures with no challenges encountered. Twenty-seven minor challenges were encountered in 20 patients. Overlap of multiple major and minor challenges was encountered in six patients. The Bonferroni test revealed age to be statistically different between the Group (I-II), Group (I-III), and Group (II-III) (Table 3).

**Table 2:** Challenges encountered in the atrial septal defect device closure in elderly patients All variables are expressed as n (%) or mean ± SD. ASD, Atrial septal defect; PAH, pulmonary hypertension; CAD, coronary artery disease; COPD, chronic obstructive pulmonary disease.

Variables	Patients (n = 37)	Group I (40–49) (n = 13)	Group I (50–59) (n = 16)	Group III (>60) (n = 13)
Total major challenges (patients)	11 (10)	4 (3)	4 (4)	3 (3)
Arrhythmia	2/11 (18.2%)	2/4 (50.0%)	0 (0.0%)	0 (0.0%)
Diastolic dysfunction	2/11 (18.2%)	1/4 (25.0%)	1/4 (25.0%)	0 (0.0%)
Significant PAH	2/11 (18.2%)	0 (0.0%)	1/4 (25.0%)	1/3 (33.3%)
Bleeding	2/11 (18.2%)	¼ (25.0%)	0 (0.0%)	1/3 (33.3%)
Stroke	1/11 (9.1%)	0 (0.0%)	1/4 (25.0%)	0 (0.0%)
Major CAD	1/11 (9.1%)	0 (0.0%)	1/4 (25.0%)	0 (0.0%)
COPD	1/11 (9.1%)	0 (0.0%)	0 (0.0%)	1/3 (33.3%)
Total minor challenges (patients)	27 (20)	9 (7)	9 (8)	9 (5)
Arrhythmia	8/27 (29.6%)	1/9 (11.1%)	4/9 (44.4%)	3/9 (33.3%)
Diastolic dysfunction	6/27 (22.2%)	3/9 (33.3%)	1/9 (11.1%)	2/9 (22.2%)
Moderate PAH	7/27 (25.9%)	3/9 (33.3%)	2/9 (22.2%)	2/9 (22.2%)
Bleeding	2/27 (7.4%)	0 (0.0%)	1/9 (11.1%)	1/9 (11.1%)
Pre-stroke	1/27 (3.7%)	0 (0.0%)	0 (0.0%)	1/9 (11.1%)
COPD	2/27 (7.4%)	2/9 (22.2%)	0 (0.0%)	0 (0.0%)
Hypertension	1/27 (3.7%)	0 (0%)	1/9 (11.1%)	0 (0.0%)
Major and minor challenge overlap patients/total number of patients	6/37 (16.2%)	2/13 (15.4%)	2/16 (12.5%)	2/8 (25.0%)
No significant challenge patients/total patients	13/37 (35.1%)	5/13 (38.5%)	6/16 (37.5%)	2/ 8(25.0%)

**Table 3:** Bonferroni post-hoc test Group I (40–49 years), Group II (50–59 years), Group III (≥ 60 years)

Variables	Post-hoc p-value		
	Group (I-II)	Group (I-III)	Group (II-III)
Age in years (Mean [SD])	<0.001	<0.001	<0.001
Gender, n (%)	0.048	0.012	0.412
Follow-up in months, (Mean [SD])	0.024	>0.999	0.353

Arrhythmia

Arrhythmia was one of the leading challenges encountered. Two patients had significant atrial arrhythmia with fast ventricular rate after the device closure causing significant palpitation. The first patient required a loading dose of amiodarone, while the second patient required a loading dose of beta-blocker to control the arrhythmia. The hospital stay of the patients was prolonged by one to two days, and they were discharged on anticoagulation and anti-arrhythmic medication. Apart from these two patients, eight other patients had atrial arrhythmias that required additional intervention in the form of intravenous adenosine and DC cardioversion during the procedure. The arrhythmia settled without any major diversion from the treatment protocol. None of these patients had pre-procedure arrhythmia documentation. Two patients in the whole cohort had atrial arrhythmia at baseline, and no challenge was encountered with these patients as they had been medically managed earlier. No patient had any evidence of complete heart block in the study.

Left ventricular diastolic dysfunction

**Figure 4:**
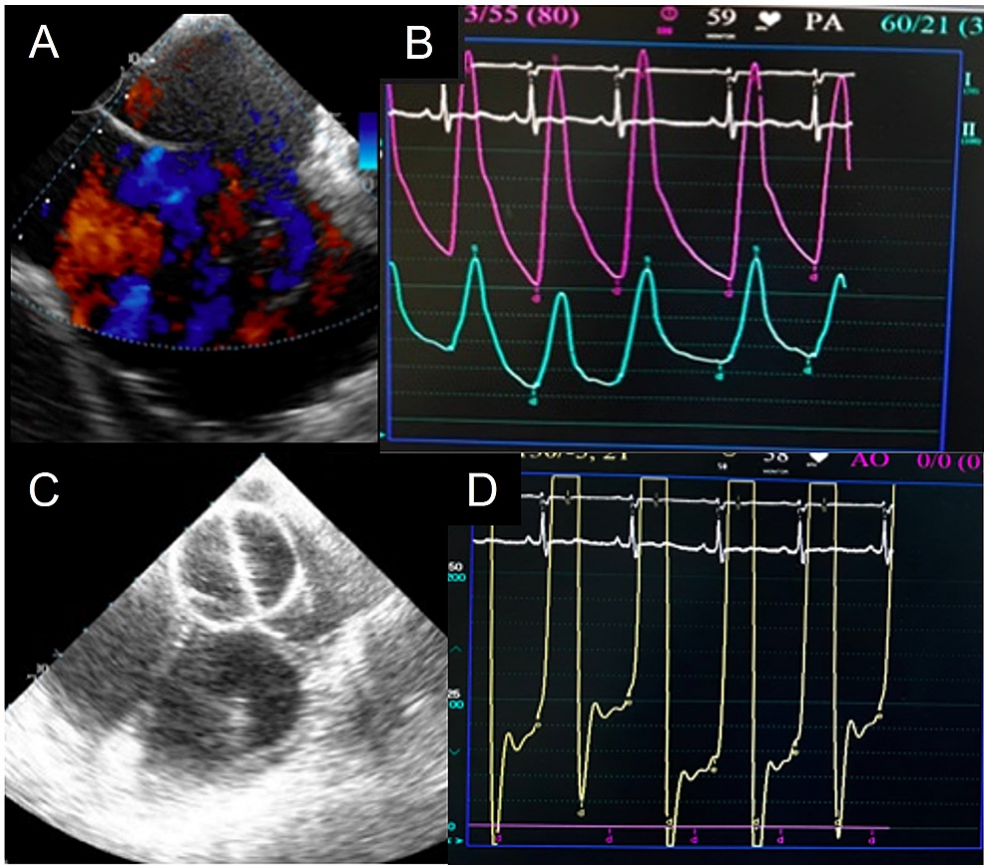
(a) Transesophageal echocardiography (TEE) with color Doppler showing large atrial septal defect with predominantly left-to-right (bidirectional shunt). (b) Basal aortic and pulmonary artery pressure. (c) TEE showing Equalizer balloon occlusion of the defect. (d) Post balloon occlusion elevated left ventricular end-diastolic pressure (50 scales).

**Figure 5:**
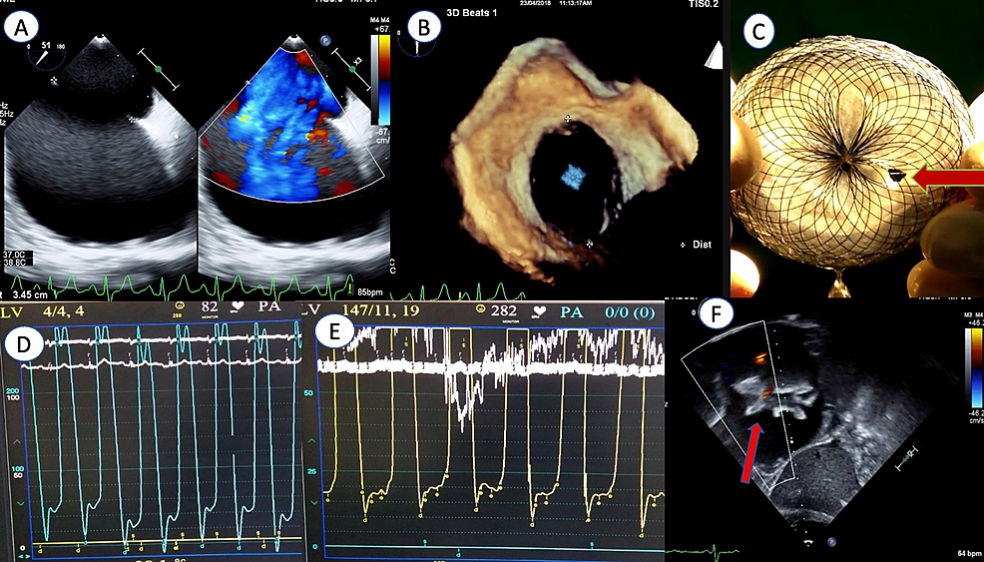
(a) Transesophageal echocardiography (TEE) with color Doppler showing very large atrial septal defect with left-to-right shunt. (b) Three-dimensional TEE large defect. (c) Fenestration in the atrial septal occluder (arrow). (d) Basal left ventricular end-diastolic pressure (100 scales). (e) Post balloon occlusion left ventricular end-diastolic pressures (50 scales). (f) Post device closure transthoracic echocardiogram in subcostal view showing good device position and fenestration flow left-to-right (arrow).

Left ventricular diastolic dysfunction was another major challenge encountered in two patients. The first patient had a large ASD, significant ventricular diastolic dysfunction, atrial fibrillation, and moderate pulmonary hypertension (PAH). On the operating table, she was found to have a moderate PAH, borderline left-to-right shunt with a ratio of pulmonary to systemic (Qp:Qs) of 1.5:1.0, and pulmonary vascular resistance index (PVRI) of 1.05 WU.m^2^. The basal LVEDP was 20 mmHg. On balloon occlusion with the 33-mm Equalizer balloon (Boston Scientific, Marlborough, MA) for 15 mins, LVEDP increased from 25 to 30 mmHg with significant symptoms of cough and respiratory distress (Figure 4). The plan for device closure was abandoned. The second patient had large ASD with moderate PAH and significant diastolic dysfunction. The left-to-right shunt ratio (Qp:Qs) was 3, and PVRI was 3.73 WU.m^2^. LVEDP was 25 mmHg at baseline and decreased to 18 mmHg on balloon occlusion. Pulmonary artery pressure also reduced to a mild level. In view of LVEDP of 18 mmHg, a 5-mm fenestration was created on a 40-mm septal occluder and was deployed. The fenestration was shunting the left-to-right post-procedure (Figure 5). She did very well during the follow-up. Six patients had LVEDP ≥ 15 mmHg, which was classified as minor challenges. They received intra-procedure intravenous furosemide, and post-device occlusion the LVEDP was reassessed. In all patients, LVEDP either remained constant or reduced. All these patients did well during the follow-up.

Pulmonary hypertension

Two patients had significant PAH that necessitated diversion in the treatment plan. One 63-year-old patient had a large 30-mm ASD and pulmonary artery systolic pressure as estimated on echocardiography as 63 mmHg. During cardiac catheterization, pulmonary systolic pressure was found higher at 75 mmHg. The patient had a significant left-to-right shunt (Qp:Qs = 3.48) and PVRI of 4.65 WU.m^2^. Post closure with a 36-mm ASD occluder, pulmonary artery systolic pressure (PASP) significantly reduced to 30 mmHg. The patient stayed an extra day in the hospital for monitoring of pulmonary artery pressure. She was doing well at the short-term follow-up.

The second patient with a large ASD had delayed presentation of symptomatic severe PAH without any earlier evidence of PAH. During the pre-procedure evaluation, the estimated PASP was 44 mmHg. The history, clinical evaluation, and chest x-ray were not suggestive of significant lung disease. During cardiac catheterization, his pulmonary artery pressure was 34/10 mmHg with a mean of 18 mmHg. He underwent ASD device closure with a 36-mm ASD occluder. Pre-discharge pulmonary pressure was within normal limits. One month post-procedure, he developed significant effort intolerance and pedal edema. The right ventricular systolic pressure measured in echocardiography was around 70 mmHg with systemic venous congestion. The symptoms were attributed to unexplained severe PAH and right ventricular dysfunction. There was no clinical indicator of any secondary cause of PAH. He was started on pulmonary vasodilators and diuretics. He improved symptomatically over the next two months. The pulmonary vasodilator was slowly weaned off titrating with pulmonary pressure over the next six months. He is doing well in short-term follow-up. Seven patients had a moderate elevation of PAP (Table 1). The mean Qp:Qs was 2.57, and the mean PVRI was 2.48 WU.m^2^. PAP was reassessed in these groups of patients, and in all patients, it decreased significantly. At the follow-up, the PAP remained within the normal level.

Bleeding

Bleeding was a major challenge in two patients. The first patient was a 43-year-old female who had atrial arrhythmia requiring anticoagulation and amiodarone. She had significant uterine bleeding one month after the procedure. This was initially thought to be due to anticoagulant use. She was on warfarin and aspirin with an international normalized ratio (INR) within targeted ranges of 1.5-2. On further evaluation, the bleeding was attributed to multiple large uterine fibroids requiring hysterectomy to control the bleeding. In another elderly patient, an old unreported lung lesion flared up and caused bleeding during extubation after anesthesia. This required one extra day of ventilation and conservative management to settle the patient before discharge. The patient had no further bleeding episodes at short-term follow-up. Two more patients had bleeding as minor challenges, one from the large sheath access site and the second one had peri-extubation bleeding. Both resolved with conservative management. After the incidence of bleeding from the access site of a large sheath, a figure of eight stitches was included in the protocol.

Stroke

A 51-year-old patient had a stroke in the middle cerebral artery territory, three weeks post ASD closure. The patient had a brief atrial arrhythmia during the procedure requiring cardioversion. She had a 24-mm ASD device implanted. She was kept on aspirin only in view of a relatively small device. She was found to be in intermittent atrial fibrillation during admission for stroke. She was treated as per the stroke protocol, and she recovered completely. After this incident, the unit policy was changed for giving either dual antiplatelet in low-risk patients or anticoagulation along with antiplatelet in high-risk patients. Patients with large ASD with large devices and suggestion of atrial arrhythmia or history of thromboembolism were considered as a high-risk population for anticoagulation therapy. One patient had a pre-existing stroke that required careful peri-procedural anticoagulation and close surveillance. The management of such patients was considered a minor challenge.

Other challenges

A 52-year-old patient was found to have double-vessel CAD. The major coronary artery involvement in the patient without ischemia symptoms came as a surprise and posed a challenge in the management. The patient was given treatment options as per the standard protocol. The patient and family members chose surgery for the treatment of coronary artery obstruction. Minor CAD was present in 10 patients. These were not counted as challenges as they had not affected the treatment protocol. One elderly lady had very high blood pressure pre-procedure. Hypertension required multiple drug titrations over a few weeks, which delayed the device closure. It was a minor challenge.

One subject with a large ASD in the seventh decade had significant effort intolerance. On evaluation, the anatomy and flow across the ASD were found not to fully explain the magnitude of the symptoms. She was found to have a significant chronic obstructive pulmonary disease (COPD). She was referred to the respiratory specialist. Appropriate respiratory treatment for four weeks with proper medications resulted in the improvement of symptoms. She was taken for ASD device closure under multidisciplinary team care. The pulmonary artery pressure in the index patient in cardiac catheterization was 31/16/mean of 21 mmHg. She had successful closure of the ASD with the device. She required continued respiratory therapy and care. The management of the patient came as a major challenge. Two additional patients who required appropriate COPD therapy were considered minor challenges.

## Discussion

Secundum ASD is one of the most common congenital heart defects in grown-up adults [1]. Device closure of ASD is a preferable intervention in patients of suitable age. Untreated secundum ASD is known for complications in older patients. The problems are usually related to long-standing left-to-right shunt and associated right heart dilatation leading to right ventricular dysfunction, atrial arrhythmia, PAH, thromboembolic episodes, and bleeding due to anticoagulation therapy given for associated atrial arrhythmia. Some complications are age-related problems such as CAD, COPD, and hypertension. The risk factors for complications are related to each other directly or indirectly. It is very important to detect and manage the risk factors at different stages of ASD intervention to minimize the complications. A thorough individualized evaluation of risk factors, potential physiological changes, and complications followed by a meticulous treatment approach for transcatheter ASD closure in older patients optimizes the outcomes [9].

Atrial arrhythmia is one of the most common complications of ASD at a late age. It is caused by chronic atrial stretch-induced modification in the structural and cellular architecture of the atrium. More than 40% increase in the atrial area is a known risk factor for the same [2,10,11]. Atrial arrhythmia has multiple cascading effects including thromboembolism in older patients with ASD. Usually, the incidence of atrial arrhythmia reduces after ASD closure due to the reduction of right atrial size and remodeling. In one group of patients, the right atrium may be persistently dilated due to chronic stretch-induced fibrotic changes. This group of patients may have a persistent atrial arrhythmia that may require therapy. The possibility of atrial arrhythmia that can be intermittent should be kept in mind at different stages of ASD closure. Failure to recognize the same can lead to complications as found in the present study. There are two approaches to deal with atrial arrhythmia. Rate control strategy with anticoagulation is an acceptable good alternative to ablation and medication [9,12]. The presence of atrial arrhythmia influences the anticoagulation and antiplatelet strategy in these patients. Older patients with a history or risk of atrial arrhythmia should be screened meticulously to identify any arrhythmia. They should be given anticoagulation therapy along with antiplatelet. The older patients having a very large (≥38 mm) device size also need to be anticoagulated in view of higher risk for clot formation. Anticoagulation therapy has its own risk as it can cause bleeding.

Complete heart block (CHB) is another type of arrhythmia seen in the elderly population, which will require permanent pacemaker implantation. Sometimes, complete heart block may coexist in a given patient with large ASD. Alternatively, a patient who had permanent pacing for CHB may be diagnosed with significant ASD subsequently. The device closure in a patient with permanent pacing or simultaneous placement of pacing lead and ASD device is challenging. It can have complications in the form of lead displacement, injury to cardiac structure, or technically, the procedure will be difficult. Careful planning and execution of the procedure under imaging and fluoroscopic guidance can help to ensure optimal outcomes [13].

Left ventricular diastolic dysfunction is a major challenge in older ASD patients undergoing device closure. The left ventricular myocardium had age-related elastic stiffness and reduced diastolic compliance. A long-standing left-to-right shunt at the atrial level changes the septal configuration, and the bowing of the interventricular septum toward the left ventricular results in under-filling of the left ventricle. ASD device closure results in a significant improvement in the right ventricular filling and function. Significant right ventricular volume reduction was seen in the first 24 hours of the device closure, which continues to improve in the first six months after the procedure. The improvement in the right ventricular function and the filling pressure results in improvement of left ventricular compliance due to ventricular interdependence. This results in improved left ventricular diastolic function, improved left ventricular isovolumetric relaxation timing, preload parameters, LV diastolic dimension, and improved left ventricular ejection fraction [10].

All the patients undergoing device closure for large ASD may not have a favorable improvement in the left ventricular diastolic function. In one group of patients, ASD device closure leads to a significant elevation of LVEDP due to the manifestation of masked ventricular restriction. The percentage of patients showing the phenomenon of significant elevation of LVEDP post device closure is varied. It ranged from 2% to 3.6% of patients as reported by Choi et al. [9] to 23.6% of patients older in 60% age in patients as seen by Abdelkarim et al. [14]. The latent left ventricular diastolic dysfunction can be assessed by baseline evaluation of LVEDP during catheterization and then occluding the ASD by an appropriate balloon or with the device itself and waiting for 15 mins. Woo et al. [15] suggested test occlusion of ASD with mean LA pressure, >15 mmHg or LVEDP more than >18-20 mmHg for 15 mins. If pressure increases by 5 mmHg after the occlusion, the device requires fenestration with a continuation of the heart failure medication. Analysis of left ventricular diastolic function is extremely important for older patients undergoing ASD device closure to prevent serious complications. The high-risk candidates with tolerably elevated LVEDP can be fenestrated [10,14,16,17]. Older people frequently associate high blood pressure. Long-standing hypertension is a known cause of significant left ventricular diastolic dysfunction. This substrate of patients should be carefully evaluated to prevent complications [18].

PAH is a known accompaniment for untreated ASD. Cherian et al. found 17% of their cohort having pulmonary arterial systolic pressure more than 50 mmHg [19]. Mild to moderate PAH is relatively common in the fifth decade and beyond. Usually, the pulmonary pressure decreases or remains steady in mild levels after the closure. Assessment of PAP by echocardiography usually is corroborative with cardiac catheterization data but may not be absolutely matching, and sometimes, surprises do happen as found in the index series. Delayed elevation was also seen in the present study. These patients require close and continued monitoring [2,9,20].

Older patients without any symptoms suggestive of CAD can have coronary artery involvement of different grades. CAD is an additional issue that needs to be resolved for patients requiring ASD closure. In addition, CAG can detect coronary abnormalities like abnormal origin or course, which can get obstructed with ASD device closure [21]. Hence, patients undergoing ASD device closure should have a CAG to delineate the coronary anatomy. Mild CAD can be addressed by medical means, whereas significant coronary artery obstructions require intervention based on the individual lesion. Discrete lesions amenable to stenting can have concurrent stenting along with ASD device closure. Some lesions may require surgery; 6% of cases in one study were found to have some coronary artery involvement with one patient requiring stenting [13,15].

Bleeding episodes are a challenge that can come as a complication of therapy or may be precipitated by a pre-existing problem. This possibility should be kept in mind when managing ASD device patients. Inadequate anticoagulation can result in thromboembolism and stroke. Thromboembolism is more common in patients with atrial fibrillation and significant PAH. A metal prosthesis is also more prone to develop clots. Thromboembolism causes cerebrovascular accidents and is a recognized complication after ASD closure, which is unrelated to the type of closure device or surgery [13,20]. The index study cohort had both types of challenges. A proactive approach in detection of the risk factors, counseling of the patients for the anticoagulation therapy, bleeding-related complications, and appropriate management of the problem can reduce the chance of complication.

Older patients beyond the fifth decade have a higher incidence of COPD. In patients with significant ASD, the symptoms are primarily attributed to the large ASD. The ASD can increase the hyperresponsiveness of the airway in up to 63% of the patients. COPD can partly contribute to pulmonary hypertension [22]. A significant reduction in the COPD symptoms was found in up to 80% of patients with initial COPD symptoms. Older subjects with ASD should be screened for respiratory elements in suspected cases and treated appropriately. This has the double benefit of symptomatic improvement and reduction in the PAH and associated challenges in device closure also found in the present study [22,23].

The device closure of ASD in older age can have multiple challenges. The increasing age from the fifth decade and beyond was not found associated with increased risk in the index study though the sample size was limited. The safety of the device closure in the late age is also reported in the literature. The risk factors in the device closure of ASD in the older are unique and linked to the prolonged left-to-right shunt and age-related. These risk factors should be thoroughly screened, and a protocol should be made to treat them proactively from the pre-intervention stage (Table 4). A clear understanding of the common risk factors that can prove troublesome in ASD intervention will help to plan the procedure better. The patient and the family can be counseled in detail about the procedure and educated about the possible complications like arrhythmia, anticoagulation therapy, and bleeding to improve the outcomes.

**Table 4:** A proposed approach for the management of challenging factors CAD, Coronary artery disease; ASD, atrial septal defect; LVEDP, left ventricular end-diastolic pressures; Qp:Qs, the ratio of pulmonary and systemic blood flow; PVRI, pulmonary vascular resistance indexed.

Challenging factors	Approach
Arrhythmia	(1) Baseline evaluation in patients with a history of palpitation and intermittent arrhythmia.
	(2) Risk assessment for arrhythmia. Patients with large defects and older patients are more at risk.
	(3) Holter monitoring in suspected subjects for arrhythmia.
	(4) Treatment of arrhythmia and anticoagulation, if recommended.
Diastolic dysfunction	(1) Detailed evaluation of diastolic function with an echocardiogram.
	(2) At-risk population approach for hypertensive subjects.
	(3) Medical treatment for diastolic dysfunction before the procedure.
	(4) Basal checking of LVEDP and pulmonary artery pressure.
	(5) Balloon/device occlusion for 15 minutes and rechecking LVEDP in patients with elevated LVEDP (>15 mmHg).
	(6) If LVEDP is elevated and increases more than 5 mmHg, fenestration of the device is recommended along with intra-procedure diuretics and continuation of medical therapy.
Pulmonary hypertension	(1) Baseline detailed clinical and imaging evaluation for pulmonary hypertension and quantum of left-to-right shunt.
	(2) Catheter assessment of pulmonary artery pressure at baseline.
	(3) Assessment of Qp:Qs and PVRI in the high-risk patients with moderate to severely elevated pulmonary artery pressure.
	(4) If device closure is done in the favorable category, post-device closure checking of pulmonary artery pressure.
	(5) Close monitoring of pulmonary artery pressure in the follow-up for a delayed manifestation of pulmonary hypertension.
Bleeding	(1) Identification of at-risk subjects with bleeding tendency, history of anticoagulant intake, or another potential cause of bleeding.
	(2) Meticulous anticoagulation monitoring peri-procedure.
	(3) Blood product requisition in high-risk cases.
	(4) Treatment of the associated cause.
Stroke/thromboembolism	(1) Identification of at-high-risk subjects, especially with arrhythmia.
	(2) Detail pre-procedure imaging to rule out the clots in high-risk cases.
	(3) Optimum antiplatelet and anticoagulation: individualized approach.
	(4) Regular surveillance.
Hypertension	(1) Pre-procedure evaluation for hypertension and treatment history of antihypertensive use.
	(2) Evaluation of the effect of hypertension in the heart and assessment of diastolic function.
	(3) Target optimal control of blood pressure even before the procedure.
	(4) Checking of LVEDP for hypertensive subjects, and if diastolic dysfunction presents, then approach like diastolic dysfunction.
Coronary artery disease	(1) Identification of patients with risk factors for coronary artery disease.
	(2) Echocardiographic delineation for any regional wall motion abnormality.
	(3) Pre-ASD closure counseling for treatment possibility of coronary artery disease on table in patients with no CAD symptoms.
	(4) Mandatory evaluation of coronary anatomy before or during ASD device closure.
	(5) Simultaneous intervention of significant coronary lesions if found in coronary angiogram suitable for transcatheter intervention.
	(6) Post-procedure medical management of the minor coronary lesion.
Chronic obstructive airway disease	(1) Identification of existing airway disease, if any, especially if symptoms are disproportionate for the atrial septal defect.
	(2) Optimal treatment for airway disease, teaming with respiratory specialists.
	(3) Vigilant intra- and post-operative care along with a respiratory team in high-risk patients.

Study limitations

The study had a few limitations. The first limitation is the small sample size due to which the study lacked the statistical power to evaluate all parameters influencing the challenges. The second and most significant limitation of this study was the retrospective study design. The third limitation is the preferential referral of older patients with larger defects to the index unit for device closure and possible selection bias as a single lead operator was involved in final decisions. Lastly, the follow-up period was relatively short. Hence, a prospective study with a larger sample size can shed more light on this topic.

## Conclusions

ASD is common in grown-up adults. The majority of secundum ASD can be closed by transcatheter intervention. Older patients who are in the fifth decade and beyond with secundum ASD have risk factors that can lead to complications at various stages of intervention. The long-standing atrial level shunt in late age gives rise to multiple challenges in the safe execution of the transcatheter intervention. The risk factors pose challenges in ASD intervention that may warrant alteration in the standard treatment protocol. Thorough screening and surveillance for the risk factors like arrhythmia, pulmonary and systemic hypertension, left ventricular diastolic dysfunction, and COPD help in doing the device closures with minimal complications. A multidisciplinary approach involving intensive care specialists, anesthesia teams, and other relevant specialists in a given patient is essential for the management of the challenges. The prevention and management of the complication should start from the pre-procedure evaluation stage to detect the high-risk category and monitor closely during the intervention and post-procedure follow-up period.
